# Methylcellulose–Alginate Composite Bead Incorporating Ethanol and Clove Essential Oil: Properties and Its Application in Bakery Products

**DOI:** 10.3390/polym17101377

**Published:** 2025-05-17

**Authors:** Jurmkwan Sangsuwan, Prem Thongchai, Kanarat Nalampang

**Affiliations:** 1 Faculty of Agro-Industry, Chiang Mai University, Chiang Mai 50200, Thailand; prem.th@cmu.ac.th; 2 Faculty of Science, Chiang Mai University, Chiang Mai 50200, Thailand; kanarat.n@cmu.ac.th

**Keywords:** alginate, methylcellulose, ethanol, clove, essential oil, vanillin, active packaging

## Abstract

Antifungal composite beads were prepared using a methylcellulose, alginate, and ethanol solution with the ionic gelation method and ethanol beads (E). A total of 1.0 mL of clove essential oil (CEO) and 1.0 g of vanillin were added to provide an antifungal effect against *Aspergillus flavus* and *Rhizopus stolonifera*. Four bead formulations were prepared: ethanol beads (E), ethanol beads containing CEO (EC), ethanol beads containing vanillin (EV), and ethanol beads containing vanillin and CEO (EVC). Ethanol beads were transparent and spherical, whereas those containing CEO or vanillin were spherical and opaque, with diameters ranging from 2.1 to 2.4 mm. The surface and pores in the polymer matrix were investigated in relation to the encapsulation and release of antifungal agents. The bursting release of ethanol and CEO occurred on the first day. Antifungal assays on potato dextrose agar against *Aspergillus flavus* and *Rhizopus stolonifera* showed that beads containing CEO (EC and EVC) provided superior inhibition, particularly at a dosage of 1.0 g. In butter cake preservation tests, packaging the butter cake with a sachet containing 1.0 g of EC or EVC beads can extend the shelf life by two days, delaying visible mold growth from day 5 to day 7 compared to the control.

## 1. Introduction

The growing consumer preference for natural food, which prioritizes items with no artificial preservatives, coupled with the necessity for prolonged shelf lives, has driven the use of plant extracts, GRAS, in the food industry. When no preservatives are used, bakery products such as bread and cake have a short shelf life of 3–5 days at room temperature, which can lead to mold growth [1]. Many plant extracts possess antimicrobial characteristics, which can be used as an alternative to chemical preservatives to prolong the shelf life of food products by suppressing the growth of microorganisms that are responsible for the deterioration of food quality. Clove extract is used to inhibit the growth of bacteria and fungi. The active component, eugenol, exhibits strong antimicrobial activity against a variety of fungi and has been used traditionally for its preservative and medicinal properties. The mode of action of eugenol is to damage the cell surfaces and cell membrane integrity of fungi, leading to the leakage of cellular contents and ultimately cell death [2]. Clove extracts are commonly used in the food and beverage industry to preserve a variety of products, including baked goods, meat products, and beverages, where fungal contamination can occur. Clove extract can also be used synergistically with other natural preservatives to enhance the overall preservation efficacy, providing a broader spectrum of protection against different microorganisms. According to the findings of Sethunga et al. [3], the combination of cinnamon bark oil and clove bud oil exhibited a synergistic effect against *Aspergillus niger* in comparison to the use of clove bud oil alone. Vanillin, a natural compound that imparts the characteristic flavor and aroma of vanilla, has been recognized for its potential antifungal properties by inhibiting the growth and development of various fungi. It can interfere with the fungal cell membrane, disrupting its integrity [4]. Vanillin can be incorporated into food products as a natural preservative, especially in items where fungal spoilage is a concern.

Sodium alginate, a natural polysaccharide derived from brown algae, is used for encapsulation purposes in the food industry. It can encapsulate flavors, colors, vitamins, and other sensitive ingredients, including volatile plant extracts, protecting them from degradation while regulating their release [5]. Additionally, methylcellulose can be used to encapsulate ethanol [6], which inhibits the development of mold and prevents the formation of aflatoxin [7]. Ethanol vapor released from ethanol pads (2 g/pad) has demonstrated efficacy in delaying microbial growth in bakery products [8]. However, current applications of encapsulated ethanol together with volatile plant extracts in biodegradable polymers for bakery preservation remain limited in both research and commercial implementation. This study investigates the development of methylcellulose–alginate composite beads designed to entrap and controllably release ethanol, clove essential oil, and vanillin, creating an active antifungal atmosphere within the packaging to extend the shelf life of bakery products. The objectives of this study were (1) to develop methylcellulose–alginate beads containing ethanol, vanillin, and clove essential oil and study their properties, and (2) to evaluate the antifungal effect of beads in vitro and in butter cake packages.

## 2. Materials and Methods

### 2.1. Materials

Methylcellulose (M0512) was acquired from Sigma-Aldrich, St. Louise, MO, USA. Ethanol was acquired from RCL Labscan, Bangkok, Thailand. Clove essential oil was acquired from Chemipan Co., Ltd., Bangkok, Thailand. Tween 20 was acquired from NOF Corporation, White Plains, NY, USA. Ethyl vanillin was acquired from Rhodiarome, Baton Rouge, LA, USA. Alginate was acquired from Qingdao Bright Moon Seaweed Group Co., Ltd., Qingdao, China.

### 2.2. Bead Formulations and Forming

Beads were prepared by dissolving 1.0 g of methylcellulose (M0512, Sigma-Aldrich, St. Louise, MO, USA) in 50 mL of 86% ethanol solution (RCL Labscan, Bangkok, Thailand). The MC solution was stirred by an overhead stirrer for 1 h at ambient temperature until the solution was clear. In the formulation with clove essential oil (Chemipan Co., Ltd., Thailand), 1.0 mL of CEO and 0.2 mL of tween 20 (NOF Corporation, USA) were added into the MC solution as antifungal agents. In addition, 1.0 g of ethyl vanillin (Rhodiarome, Baton Rouge, USA) and 0.75 g of alginate (Qingdao Bright Moon Seaweed Group Co., Ltd.) were dissolved in 50 mL of distilled water. Both solutions were mixed by a mechanical stirrer for 1 h at ambient temperature until the solution was homogeneous. The bead-forming solution was added into a 25 mL syringe with a 1.2 × 25 mm Nipro needle (Nipro, Mechelen, Belgium). Then, the beads were obtained dropwise by mixing the solution in a 2.0% calcium chloride solution. After 30 min, the beads were filtered and washed with distilled water 5 times. The beads were kept in a hermetically sealed glass vial at 25 ± 2 °C before evaluation. There were 4 bead formulations: (1) methylcellulose–alginate beads (E), which trap only ethanol, (2) methyl cellulose–alginate beads containing ethanol and clove essential oil (EC), (3) methylcellulose–alginate beads containing ethanol and vanillin (EV), and (4) methylcellulose–alginate beads containing ethanol, vanillin, and clove essential oil (EVC).

### 2.3. Analysis of Essential Oils 

The compositions of vanillin and CEO were analyzed using gas chromatography techniques (7890A Agilent Technology, Santa Clara, CA, USA). An MSD 5975C (EI) mass spectrometer was used as the detector, with conditions according to former work [9].

### 2.4. Bead Properties

#### 2.4.1. Determination of Size and Appearance

The bead diameter was measured using a Vernier caliper. The reported diameter values were the average of 50 beads from each treatment. The appearance of the beads was observed with the naked eye.

#### 2.4.2. Determination of Fourier-Transform Infrared (FT-IR) Spectroscopy

Beads were analyzed using a Fourier-transform infrared (FT-IR) spectroscopy method with attenuated total reflectance (Jasco, FTIR-4700, Tokyo, Japan). The scan condition was set with a resolution of 4 cm^−1^ and a range of 4000–600 cm^−1^. The beads were determined either when wet or were dried in a hot air oven at 105 °C for 24 h.

#### 2.4.3. Surface Determination

After the beads were dried in a hot air oven at 105 °C for 24 h, the surfaces of the beads were characterized using a scanning electron microscope (SEM Joel JSM-IT800, Tokyo, Japan).

#### 2.4.4. Pore Determination

The surface area, average pore diameter, and pore volume of dried beads were measured with respect to nitrogen adsorption–desorption isotherms using a surface area and pore size analyzer (Quantachrom, model autosorb1 MP, Anton Paar QuantaTec Inc., Boynton Beach, FL, USA). Samples were degassed at 120 °C for 8–15 h under vacuum. The specific surface area of the samples was calculated according to the Brunauer–Emmett–Teller (BET) method. In addition, the volume of the mesopores was calculated according to the Barrett–Joyner–Halenda method.

### 2.5. Encapsulation and Release of Ethanol

The amount of ethanol loaded in the beads was determined by gas chromatography (Agilent 7890B, MA, USA) using an HP-5 column (Agilent, MA, USA) with dimensions of 30 m × 0.25 mm × 0.25 µm. The 1.0 g beads were placed in a 20 mL vial and sealed with a PTFE/Si septum (Sigma-Aldrich, St. Louise, MO, USA). The vial was incubated at 80 °C for 5 min. The gas (1000 µL) was withdrawn from the vial. The conditions were set as follows: injection temperature of 220 °C, split ratio of 20:1, flow rate of 2.7226 mL/min, using helium gas as the carrier, and the column oven was started from 60 °C for 1 min and increased to 130 °C (0.5 min), with a heating rate of 20 °C/min.

The 1.0 g beads were placed in a 4 × 2.5 cm permeable cellulose bag and then placed in a 10 × 15 × 6 cm PET clamshell. The samples were taken at time intervals of 0, 3, 6, 12, 24, 48, 72, and 96 h to determine the amount of ethanol left in the beads using a standard curve. The amount of ethanol released was calculated using the following equation.

$$\%\mathrm{released}\;\mathrm{of}\;\mathrm{EtOH}=\frac{\left({EtOH}_{0}-{EtOH}_{t}\right)}{{EtOH}_{0}}\times 100$$

where ${EtOH}_{0}$ is the amount of ethanol entrapped in the beads and ${EtOH}_{t}$ is the amount of ethanol remaining in the beads at a specific time.

### 2.6. Encapsulation and Release of CEO and Vanillin

The amounts of CEO or vanillin loaded in the beads were determined using a UV–vis spectrophotometer (Jasco V730, Japan). The analysis method was mainly adapted from Hosseini et al. [10], with some modifications. The beads (0.2 g) were drenched in 20 mL of 75% ethanol for 90 min using an ultrasonic bath (Daihan Scientific Co. Ltd., Wonju-si, Republic of Korea) at 80 °C to extract the oil from the beads. Then, the CEO or vanillin, which were dissolved in the ethanol, were subjected to measure the UV absorption at wavelengths of 280 nm for clove and 230 nm for vanillin. The encapsulation efficiency of the beads was calculated as the percentage of CEO or vanillin encapsulated with respect to the initial amount of CEO or vanillin used, as shown in the following equation:

$$\%\mathrm{EE}=\frac{A_{0}}{A}\times 100$$

where $A_{0}\;$ is the amount of CEO or vanillin entrapped in the beads after production and A is the amount of CEO or vanillin in the bead formulation.

The beads (0.2 g) were placed in a 4 × 2.5 cm permeable cellulose bag and then placed in a 10 × 15 × 6 cm PET clamshell. The samples were taken at time intervals (0, 24, 48, 72, and 96 h) and drenched in 20 mL of 75% ethanol for 90 min using an ultrasonic bath (Daihan Scientific Co. Ltd., Korea) at 80 °C to dissolve the remaining CEO or vanillin in the beads. To analyze the concentration of CEO or vanillin in the beads, the ethanol used for extraction was subjected to measurements of absorbance at wavelengths of 280 and 230 nm using UV–vis spectroscopy. The release of the CEO or vanillin with respect to time was determined as follows:

$$\%\mathrm{released}\;\mathrm{of}\;\mathrm{EOs}=\frac{\left(E_{0}-E_{t}\right)}{E_{0}}\times 100$$

where $E_{0}\;$ is the amount of CEO or vanillin entrapped in the beads and $E_{t}$ is the amount of CEO or vanillin remaining in the beads at a specific time.

### 2.7. Isolation of Aspergillus flavus and Rhizopus stolonifera

*Aspergillus flavus* and *Rhizopus stolonifera* were isolated from moldy bread according to a previous method [9]. A small portion of the mold was transferred onto the agar plate using a sterile needle and incubated at 25 °C. The mycelia were transferred to a new agar plate for further purification and were identified under a microscope to obtain purified *Aspergillus flavus* and *Rhizopus stolonifera*.

### 2.8. Antifungal Effect of Beads Against Aspergillus flavus and Rhizopus stolonifera on Potato Dextrose Agar

The mycelium of *Aspergillus flavus* and *Rhizopus stolonifera* was cultured on PDA using a sterile needle, placing 4 points 3 cm apart from the center. Each formulation’s beads (0.5 g and 1.0 g) were placed in small sterile cups and positioned at the center of solidified PDA. All Petri dishes were incubated at 25 °C. The total areas of mold growth in each plate were measured daily for 4 days for *Aspergillus flavus* and 3 days for *Rhizopus stolonifera*.

### 2.9. Shelf Life of the Butter Cake Packed with Beads

The preservative-free butter cake (8.4 × 8 × 1.5 cm) had a moisture content (MB120 Moisture analyzer, OHAUS, Parsippany, NJ, USA) of 28.45 ± 1.19% and water activity (AquaLab Pre, METER Group, Inc., Pullman, WA, USA) of 0.904 ± 0.00. Each slice of butter cake was individually packed in a PET 1000 mL clamshell (15 × 10 × 6.5 cm), containing 1.0 g of E, EC, EV, and EVC beads packed in a permeable sachet (4 × 2.5 cm). A butter cake in a clamshell without beads served as the control. All treatments were stored at 25 ± 1 °C for 7 days, with daily observation for visible mold growth.

The texture (hardness) of the butter cake was measured on days 1, 3, 5, and 7 using a TA.XTplusC texture analyzer (Stable Micro Systems, Surrey, UK). The parameters set for bread are a pre-test speed of 1.0 mm/s, test and post-test speeds of 2.00 mm/s, target mode: strain, strain: 40%, time: 5 s, trigger type: auto (force), and trigger force: 5 g. The probe used was a cylindrical aluminium probe with a diameter of 36 mm.

#### Statistical Analysis

Data were subjected to an analysis of variance, with a completely randomized design. Means comparisons were tested using Tukey’s b multiple-range (α = 0.05) test using SPSS version 11.0 (SPSS Inc., Chicago, IL, USA) software.

## 3. Results and Discussion

### 3.1. Composition of Vanillin and Clove Essential Oil 

CEO is mainly composed of isopropyl tetradecanoate (50.82%), eugenol (41.94%), and caryophyllene (4.04%), while vanillin comprises 4-hydroxy-3-methoxy-benzaldehyde (98.13%).

### 3.2. Bead Properties

#### 3.2.1. Size and Appearance

**Figure 1:**
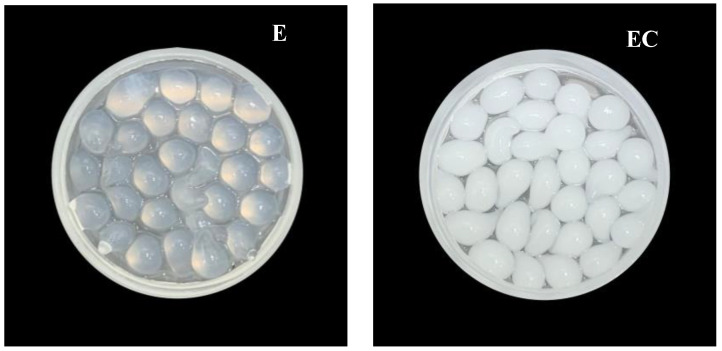

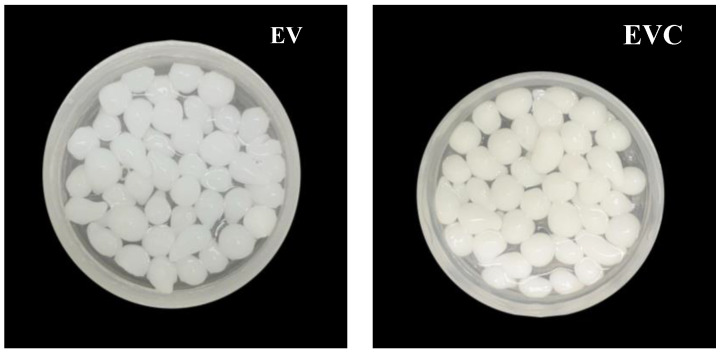
Appearance of E, EC, EV, and EVC beads.

All the developed beads are spheres. The diameters of ethanol beads (E), ethanol beads containing CEO (EC), ethanol beads containing vanillin (EV), and ethanol beads containing clove and vanillin (EVC) were 2.2 ± 0.5, 2.4 ± 0.5, 2.1 ± 0.3, and 2.2 ± 0.5 mm, respectively. The appearance of the beads is illustrated in Figure 1. The E beads are transparent spheres, whereas the EC, EV, and EVC beads, which contain CEO and vanillin, are opaque due to light reflection and dispersal caused by the presence of oil droplets. Zhang et al. [11] also observed less transparency in sodium alginate films containing ginger EO, compared with films without EO. Essential oils are typically hydrophobic and do not mix well with water.

#### 3.2.2. Fourier-Transform Infrared (FT-IR) Spectroscopy

**Figure 2:**
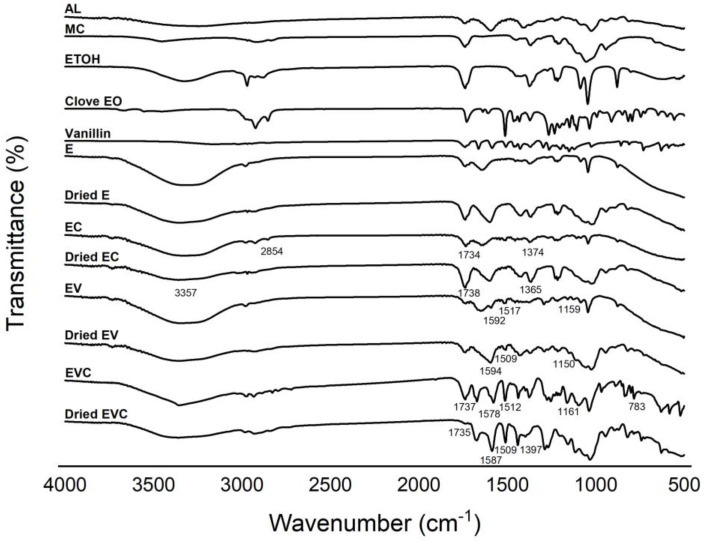
FTIR spectra of alginate (AL), methylcellulose (MC), ethanol (ETOH), clove EO, vanillin, E, EC, EV, and EVC beads.

The FT-IR spectra of the prepared methylcellulose–alginate composite are shown in Figure 2. Obviously, all spectra of polymer beads that contain methylcellulose–alginate show the presence of -OH stretching, with a broad peak at around 3700–3100 cm^−1^. In addition, the -CH stretching region of the aromatic is shown between 3000–2800 cm^−1^. An interesting peak was noticed at 1592 cm^−1^ from pure alginate, which is assigned to the acyl group. Since clove oil was added to the polymer beads, aromatic -CH stretching was observed in every sample. These results are in good agreement with previous works [12,13]. Moreover, in every dried polymer bead that contained clove oil, this specific -CH aromatic stretching can be noticed. This indicates that clove oil incorporates well into the structure of methylcellulose–alginate composites. In addition, the polymer beads with vanillin showed IR spectra with the vibration of benzene at 1592 cm^−1^ and 1517 cm^−1^, corresponding to the C=C stretching vibrations within the aromatic ring, together with C-O-C stretching at 1159 cm^−1^ for both wet and dried beads, which agrees with Shekarforoush et al. [14]. This probably assumes that adding clove oil and vanillin into methylcellulose–alginate composites could be employed in bakery applications.

#### 3.2.3. The Surfaces and Pores of Beads

**Figure 3:**
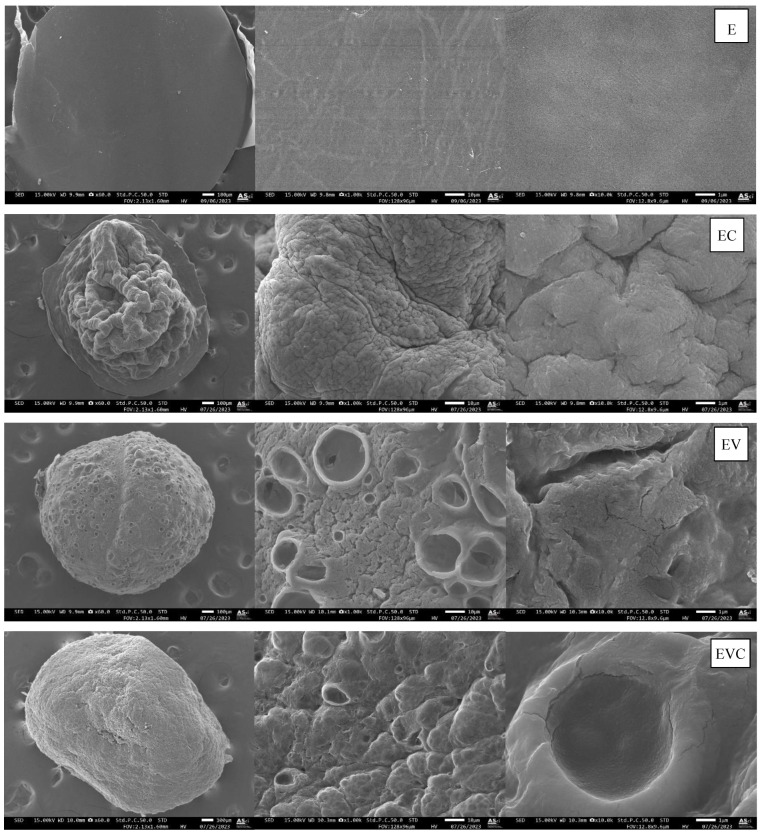
Surface of E, EC, EV, and EVC beads at 60× 1 kx and 10 kx.

The surfaces of dried beads are shown in Figure 3. The ethanol bead (E) has a smooth surface. Beads containing CEO (EC) showed shrinkages on the surface, but no pore was observed, while beads containing vanillin (either EV or EVC) had rough surfaces with varying sizes of holes throughout the surface area. Rezvanian et al. [15] reported the micrograph of a pure sodium alginate film and revealed that its surface was dense and devoid of any cavities due to ionic crosslinking. The calcium ion interacted with the guluronate block of alginate, thereby stimulating the chain’s combination and the formation of egg-box junctions. The addition of CEO and vanillin resulted in a rougher surface because volatile substances evaporated during the drying process, leaving gaps in the surface of the beads.

The pore diameter, specific surface, and pore volume of dried beads are detailed in Table 1. Ethanol beads had the largest pore diameter, followed by EV, EVC, and EC. The pore size or diameter had an inverted relationship with the specific surface and pore volumes. In this case, the methylcellulose–alginate matrix containing solely ethanol had a larger pore size, resulting in the smallest specific surface and pore volumes. Pores in ethanol beads are located inside the bead bulks since they were not observed in SEM micrographs. While beads containing vanillin and/or CEO had smaller pores throughout the methylcellulose–alginate matrix. Klangmuang and Sothornvit [16] also demonstrated that the incorporation of plai and ginger EOs into nanocomposite films based on hydroxypropyl methylcellulose (HPMC) resulted in the dispersion of sponge-like cavities and loose structures throughout the HPMC matrix.

### 3.3. Encapsulation and Release of Ethanol

**Figure 4:**
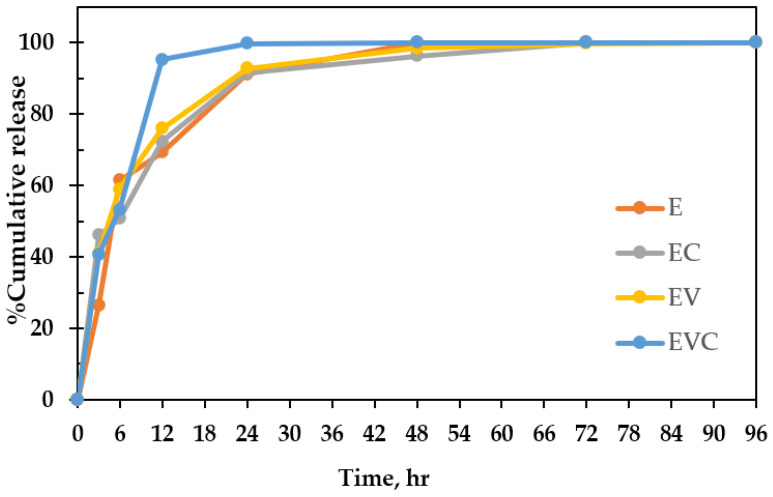
Percentage cumulative release of ethanol from E, EC, EV, and EVC beads.

This research focuses on the synergistic antimicrobial effect of ethanol and plant extracts (CEO and vanillin) in comparison to their individual applications. Presently, ethanol is extensively utilized in the bakery industry, having obtained generally recognized as safe (GRAS) status. Nevertheless, the practical implementations of ethanol emitters were constrained due to their rapid volatilization and unregulated discharge rates [17]. Consequently, using the methylcellulose–alginate matrix to regulate the release of ethanol, CEO, and vanillin was investigated. Methylcellulose plays an important role in trapping ethanol, and alginate is used to form the beads. The ethanol content within the methylcellulose–alginate matrix was measured both immediately after bead formation (0 h) and at specified time points during the release study, as presented in Table 2. Encapsulation of ethanol in E, EC, EV, and EVC beads occurred at rates of 51.8%, 57.1%, 32.0%, and 57.9%, respectively. EVC beads had the highest encapsulation efficiency (279.02 ± 29.49 mg ethanol/g bead), followed by EC, E, and EV beads. At 24 h, EVC beads also released ethanol at the fastest rate, and had almost no ethanol left. This might be because of the pore at the bead’s surface (Figure 3), which causes the ethanol encapsulated in the bead to be released very quickly, compared to E and EC beads, which had a smooth surface without holes. The faster release of ethanol from beads occurred in the first 12 h and slowed down afterward. The release of ethanol from ethanol beads (E) is complete within 48 h. Other bead formulas had a slower release rate. Ethanol in EVC beads was completely released after 72 h, while in EC and EV beads, it was completely released after 96 h (Figure 4). The release of ethanol from each bead formulation based on the R^2^ values of kinetic models is demonstrated in Table 3. All bead formulations showed the best fit with the Korsmeyer–Peppas model. E, EC, and EV beads had *n* < 0.43, with Fickian diffusion with minor polymer relaxation, while EVC beads had *n* = 0.54 (non-Fickian diffusion, which indicates both diffusion and polymer relaxation). The rapid release (95% in 12 h) suggests a porous or weakly crosslinked alginate matrix. This result agreed with an alginate–chitosan matrix incorporated with lavender and mentha essential oils [18].

### 3.4. Encapsulation and Release of CEO and Vanillin

**Figure 5:**
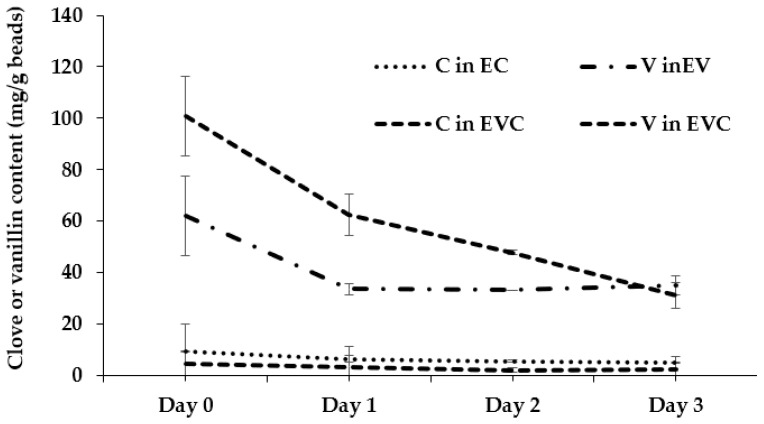
Release of clove or vanillin from beads.

The addition of CEO and vanillin is intended to synergize with the antifungal effect of ethanol vapor, which is designed to delay the development of mold [19]. Furthermore, vanillin also serves to mask ethanol’s flavor. The encapsulation and release of CEO and vanillin from each bead formula are demonstrated in Table 4 and Figure 5. The encapsulation capacity of CEO in EC beads is greater than in EVC beads due to EC beads having a greater pore volume than EVC beads. Similarly, the amount of vanillin encapsulated in EVC beads is greater than in EV beads for the same reason. The release of vanillin occurred quickly on the first day, then slowed down afterward, which may be due to the presence of free active agents on the bead surface, as they can easily diffuse into the environment [20], while CEO was released more slowly. This agreed with Sangsuwan and Sutthasupa [21], who reported that alginate beads encapsulated more CEO than CEO/vanillin.

### 3.5. Antifungal Effect of Beads Against Aspergillus flavus and Rhizopus stolonifera on Potato Dextrose Agar

The antifungal effects of beads against *A. flavus* and *R. stolonifera* are demonstrated in Table 5 and Table 6, respectively. All bead formulas can inhibit the growth of both fungi. In the control plate (no beads) on day 4, *A. flavus* in the control plate grew until it covered the media, while the plate with 1.0 g of EC, EVC, and EV beads inhibited the growth of *A. flavus* better than ethanol beads (E) without plant extracts. It is evident that the CEO and vanillin synergized the antifungal efficacy of the ethanol beads. In addition, the amount of beads used also affects the inhibition efficacy, as using 1.0 g of beads inhibited both strains better than using 0.5 g of beads. EC beads at 1.0 g released more CEO than EVC beads and appeared to offer superior inhibition against *A. flavus*, even though the two are not significantly different. Pandey [22] reported that clove at 1.5–5.0 µL/mL can inhibit *A. flavus* because clove contains eugenol, providing an antifungal effect. Luesuwan et al. [23] also reported that clove essential oil at 0.5–5% provided the highest efficiency in inhibiting *A. flavus* when compared with other types of essential oils. Furthermore, Li et al. [4] reported that vanillin at 75 µg/mL can completely inhibit *A. flavus* in 72 h on Sabouraud’s dextrose agar.

Compared with *A. flavus*, *R. stolonifera* grew more quickly, and all beads had less of an inhibitory impact on it. On day 3, *R. stolonifera* expanded until the mycelia covered the media in the control plate. All beads, aside from 0.5 g EV beads, can suppress the mycelia’s expansion. This could be due to the lower concentration of ethanol in beads (Table 2), as well as the low volatility of vanillin. The use of 1.0 g of beads significantly delayed the growth of *R. stolonifera*, compared with the use of 0.5 g. One gram of EVC and EC beads provided the best inhibition against *R. stolonifera*. For *R. stolonifera*, beads containing only ethanol were not as effective as beads containing ethanol and CEO. Therefore, CEO exhibited a superior inhibition efficacy against *R. stolonifera* in comparison to ethanol. The outcomes from this study relate to the findings of Sukatta et al. [24], who reported that clove vapor completely inhibits *R. stolonifera*, *A. niger*, and *Alternaria alternat*.

### 3.6. Shelf Life of Butter Cake Packed with Beads

#### 3.6.1. Incidence of Mold

Research on antimicrobial packaging is extensively applied to preserve bakery goods. In addition, ethanol is well-known as a preservative with antimicrobial activity. It can be sprayed directly onto the product or packaging and can also be applied inside the package in the form of ethanol emitters [25]. In this study, the antifungal effect of the beads was tested with preservative-free butter cake, which typically has a shelf life of 3–4 days and deteriorates due to mold growth on the food surface. A bead sachet containing 0.5 or 1.0 g of beads was packed with butter cake in a PET clamshell. Mold spots were visually observed and counted every day. Table 7 represents the average number of visible mold spots on the butter cake studied. It was found that, on day 5, mold growth was first observed on the control butter cake (no beads) and the butter cake packed with 0.5 g of ethanol beads (E), respectively, while mold growth on butter cakes packed with 1.0 g of ethanol beads (E) and those packed with beads containing plant extracts was delayed for 1 day. Butter cakes packed with beads containing vanillin (EV) had visually observed mold on day 6. In this case, 1.0 g of EV beads still provided a better effect on the number of mold spots, with 10-fold fewer spots than the use of 0.5 g EV beads. In addition, the treatments with 1.0 g beads containing CEO, including EC 1.0 g and EVC 1.0 g, could delay visible mold growth for another day (from day 6 to day 7). It can be concluded that all bead formulas can delay mold growth and thus extend the storage life of butter cake. On day 7, thirty-eight mold spots were observed on butter cake packed with 1 g of E beads, while only three, six, and nine spots were found on those packed with 1 g of EC, EV, and EVC beads, respectively. It is evident that CEO and vanillin synergized the inhibition effect of ethanol alone. This might be due to the ethanol being completely released from beads in one day, while CEO and vanillin continued to be released afterward. Butter cakes packed with 1.0 g of EC and EVC beads were free from mold until day 7; therefore, the shelf life of butter cake can be extended from 4 days to 6 days. Malathy et. al. [26] extended the shelf life of pita bread by mixing CEO into the bread formula and reported that 15 µL of CEO can inhibit *Rhizopus* and *Aspergillus* in pita bread for 6 days compared to the control pita bread, which had a shelf life of only 3 days.

#### 3.6.2. Texture

The hardness values of butter cake slightly increase with time during storage (Table 8). Butter cakes packed with beads in all treatments were not significantly different from the control butter cake. Therefore, the volatile substances, including ethanol, CEO, and vanillin, did not affect the texture of butter cake. Despite this, Axel et al. [27] noted that ethanol had preservative effects on bakery products.

## 4. Conclusions

Methylcellulose–alginate beads were developed to encapsulate and release ethanol, clove essential oil, and vanillin to inhibit *A. flavus* and *R. stolonifera*. The efficacy of encapsulation is correlated with the pore capacity of beads, whereas the large holes on the surfaces of the beads resulted in rapid release. The efficacy of mold inhibition depends on the amount of active substances released and the amount of beads used. All bead formulas released ethanol, which can inhibit *A. flavus* and *R. stolonifera*. Inhibition ability was improved by the incorporation of CEO and vanillin, despite the fact that CEO is more effective than vanillin due to its volatility. In addition, when butter cake is packed with a sachet containing 1 g of EC or EVC beads, mold spots appeared on day 7, whereas in the control group, mold growth was observed as early as day 5. Therefore, they can be used as an alternative method to extend the storage life of bakery products.

## Figures and Tables

**Table 1:** Pore diameter (nm), specific surface (m^2^/g), and pore volume (cc/g) of dried E, EC, EV, and EVC beads.

Bead Formula	Pore Diameter (nm)	Specific Surface (m^2^/g)	Pore Volume (cc/g)
E	870	0.975	2.122 × 10^−2^
EC	89.6	19.743	4.422 × 10^−2^
EV	674	1.437	2.423 × 10^−2^
EVC	246	5.419	3.352 × 10^−2^

**Table 2:** Ethanol content in E, EC, EV, and EVC beads after bead formation (0 h) and during the release study.

Time (hours)	Ethanol Content (mg)			
	E	EC	EV	EVC
0	204.15 ± 4.21 ^a^	237.44 ± 39.59 ^a^	162.12 ± 35.77 ^a^	279.02 ± 29.49 ^a^
3	150.07 ± 2.23 ^b^	127.96 ± 3.40 ^b^	95.73 ± 6.85 ^b^	165.52 ± 5.42 ^b^
6	78.62 ± 4.54 ^c^	117.07 ± 34.12 ^b^	66.46 ± 22.11 ^c^	130.78 ± 4.88 ^c^
12	62.61 ± 2.39 ^d^	65.68 ± 8.77 ^c^	38.84 ± 1.83 ^cd^	13.19 ± 0.28 ^d^
24	18.00 ± 3.55 ^e^	20.12 ± 2.30 ^cd^	11.70 ± 1.79 ^d^	0.77 ± 0.11 ^e^
48	0.00 ± 0.00 ^f^	8.96 ± 1.63 ^d^	2.65 ± 0.45 ^e^	0.10 ± 0.04 ^f^
72	0.00 ± 0.00 ^f^	2.30 ± 0.11 ^e^	0.27 ± 0.24 ^f^	0.00 ± 0.00 ^f^
96	0.00 ± 0.00 ^f^	0.00 ± 0.00 ^f^	0.05 ± 0.00 ^f^	0.00 ± 0.00 ^f^

Note: Different superscripts in the same column represent significant differences (*p* ≤ 0.05).

**Table 3:** R^2^ coefficients of each release model of E, EC, EV, and EVC beads.

Model	R^2^ Coefficients			
	E	EC	EV	EVC
Zero order	0.847	0.789	0.852	0.658
First order	0.932	0.901	0.945	0.874
Higuchi	0.876	0.948	0.981	0.912
Korsmeyer–Peppas	0.963	0.982	0.982	0.995

**Table 4:** Clove and vanillin content in beads.

Formula	Clove or Vanillin Content (mg/g Bead)			
	Day 0	Day 1	Day 2	Day 3
C in EC	9.35 ± 0.12 ^c^	6.42 ± 1.28 ^c^	5.47 ± 0.34 ^c^	5.05 ± 0.21 ^b^
C in EVC	4.59 ± 0.57 ^d^	3.35 ± 0.53 ^d^	2.08 ± 0.01 ^d^	2.09 ± 0.64 ^c^
V in EV	62.14 ± 15.4 ^b^	33.58 ± 2.12 ^b^	33.05 ± 0.01 ^b^	34.84 ± 3.63 ^a^
V in EVC	100.83 ± 15.46 ^a^	62.65 ± 8.06 ^a^	47.82 ± 0.88 ^a^	31.02 ± 5.11 ^a^

Note: Different superscripts in the same column represent significant differences (*p* ≤ 0.05).

**Table 5:** Area of *Aspergillus flavus* on potato dextrose agar under the atmosphere of different types and amounts of beads at 25 °C.

Bead Formula	Area of *Aspergillus flavus* (cm^2^)			
	Day 1	Day 2	Day 3	Day 4
Control	1.16 ± 0.26 ^c^	6.37 ± 0.54 ^d^	11.43 ± 2.61 ^d^	13.95 ± 1.26 ^d^
E 0.5 g	0.49 ± 0.23 ^b^	1.75 ± 1.02 ^ab^	2.64 ± 1.78 ^ab^	3.23 ± 0.77 ^b^
E 1 g	0.47 ± 0.03 ^b^	2.37 ± 0.99 ^b^	3.19 ± 0.88 ^b^	3.15 ± 0.89 ^b^
EC 0.5 g	0.65 ± 0.34 ^b^	3.04 ± 2.20 ^c^	3.80 ± 1.65 ^b^	3.86 ± 1.29 ^b^
EC 1 g	0.05 ± 0.05 ^a^	0.23 ± 0.09 ^a^	0.26 ± 0.07 ^a^	0.86 ± 0.32 ^a^
EV 0.5 g	0.58 ± 0.04 ^b^	3.75 ± 0.35 ^c^	6.18 ± 1.07 ^c^	8.16 ± 2.17 ^c^
EV 1 g	0.10 ± 0.06 ^a^	0.70 ± 0.40 ^a^	1.42 ± 1.05 ^ab^	1.81 ± 0.92 ^ab^
EVC 0.5 g	0.50 ± 0.10 ^b^	3.26 ± 0.40 ^c^	4.83 ± 0.83 ^b^	7.36 ± 1.07 ^c^
EVC 1 g	0.07 ± 0.09 ^a^	0.32 ± 0.17 ^a^	0.77 ± 0.20 ^a^	1.60 ± 0.50 ^ab^

Note: Different superscripts in the same column represent significant differences (*p* ≤ 0.05).

**Table 6:** Area of *Rhizopus stolonifera* on potato dextrose agar under the atmosphere of different types and amounts of beads at 25 °C.

Bead Formula	Area of *Rhizopus stolonifera* (cm^2^)		
	Day 1	Day 2	Day 3
Control	0.98 ± 0.16 ^e^	8.07 ± 1.21 ^e^	16.88 ± 5.55 ^d^
E 0.5 g	0.73 ± 0.11 ^d^	6.74 ± 1.18 ^d^	13.27 ± 3.23 ^c^
E 1 g	0.51 ± 0.06 ^bc^	3.85 ± 0.80 ^b^	10.30 ± 3.20 ^b^
EC 0.5 g	0.61 ± 0.14 ^c^	5.75 ± 0.31 ^c^	14.12 ± 3.19 ^c^
EC 1 g	0.36 ± 0.10 ^ab^	2.90 ± 0.90 ^ab^	5.10 ± 1.34 ^a^
EV 0.5 g	0.50 ± 0.13 ^bc^	5.94 ± 0.42 ^c^	17.08 ± 2.50 ^d^
EV 1 g	0.41 ± 0.09 ^b^	2.81 ± 0.45 ^ab^	10.23 ± 1.83 ^b^
EVC 0.5 g	0.61 ± 0.21 ^cd^	4.33 ± 1.54 ^bc^	11.94 ± 1.98 ^bc^
EVC 1 g	0.23 ± 0.03 ^a^	1.67 ± 0.91 ^a^	4.44 ± 0.01 ^a^

Note: Different superscripts in the same column represent significant differences (*p* ≤ 0.05).

**Table 7:** Mold spots on butter cake packed with different types and amounts of beads and stored at 25 °C for 7 days.

Bead Formula	Mold Growth (Spots)			
	Day 0–4	Day 5	Day 6	Day 7
Control	0 ^a^	14.4 ± 5.2 ^c^	104.6 ± 14.7 ^d^	186.0 ± 16.7 ^d^
E 0.5 g	0 ^a^	0.60 ± 0.55 ^b^	26.2 ± 5.3 ^c^	72.8 ± 17.0 ^bc^
E 1 g	0 ^a^	0 ^a^	8.4 ± 2.3 ^b^	38.4 ± 11.4 ^b^
EC 0.5 g	0 ^a^	0 ^a^	26.2 ± 3.7 ^c^	53.8 ± 11.9 ^b^
EC 1 g	0 ^a^	0 ^a^	0 ^a^	3.0 ± 1.2 ^a^
EV 0.5 g	0 ^a^	0 ^a^	30.6 ± 6.2 ^c^	63.4 ± 18.6 ^c^
EV 1 g	0 ^a^	0 ^a^	2.4 ± 0.9 ^ab^	5.6 ± 2.3 ^a^
EVC 0.5 g	0 ^a^	0 ^a^	5.0 ± 1.0 ^ab^	46.4 ± 12.2 ^b^
EVC 1 g	0 ^a^	0 ^a^	0 ^a^	9.4 ± 3.8 ^a^

Note: Different superscripts in the same column represent significant differences (*p* ≤ 0.05).

**Table 8:** Hardness of butter cake packed with 1.0 g of beads during storage.

Bead Formula	Force (kN)			
	Day 1	Day 3	Day 5	Day 7
Control	1.7 ± 0.02 ^a^	1.7 ± 0.02 ^a^	1.9 ± 0.05 ^a^	1.9 ± 0.05 ^a^
E	1.5 ± 0.02 ^a^	1.6 ± 0.07 ^a^	1.7 ± 0.04 ^a^	1.8 ± 0.08 ^a^
EC	1.5 ± 0.02 ^a^	1.6 ± 0.06 ^a^	1.8 ± 0.07 ^a^	1.9 ± 0.03 ^a^
EV	1.5 ± 0.12 ^a^	1.7 ± 0.02 ^a^	1.7 ± 0.29 ^a^	1.8 ± 0.09 ^a^
EVC	1.5 ± 0.14 ^a^	1.6 ± 0.10 ^a^	1.7 ± 0.24 ^a^	1.8 ± 0.15 ^a^

Note: Different superscripts in the same column represent significant differences (*p* ≤ 0.05).

## Data Availability

Data are contained within the article.

## Abbreviations

The following abbreviations are used in this manuscript:

CEO	Clove essential oil
E	Methylcellulose–alginate beads containing ethanol
EC	Methylcellulose–alginate beads containing ethanol and clove essential oil
EV	Methylcellulose–alginate beads containing ethanol and vanillin
EVC	Methylcellulose–alginate beads containing ethanol, vanillin, and clove essential oil
EE	Encapsulation efficiency
